# Human-Derived H3N2 Influenza A Viruses Detected in Pigs in Northern Italy

**DOI:** 10.3390/v17091171

**Published:** 2025-08-27

**Authors:** Laura Soliani, Ada Mescoli, Irene Zanni, Laura Baioni, Giovanni Alborali, Ana Moreno, Silvia Faccini, Carlo Rosignoli, Giorgia De Lorenzi, Laura Fiorentini, Camilla Torreggiani, Benedetta Cordioli, Alice Prosperi, Andrea Luppi, Chiara Chiapponi

**Affiliations:** 1 WOAH Reference Laboratory for Swine Influenza, Istituto Zooprofilattico Sperimentale della Lombardia e dell’Emilia Romagna (IZSLER), 25124 Brescia, Italy; ada.mescoli@izsler.it (A.M.); irene.zanni@izsler.it (I.Z.); laura.baioni@izsler.it (L.B.); giovanni.alborali@izsler.it (G.A.); anamaria.morenomartin@izsler.it (A.M.); silvia.faccini@izsler.it (S.F.); carlo.rosignoli@izsler.it (C.R.); giorgia.delorenzi@izsler.it (G.D.L.); laura.fiorentini@izsler.it (L.F.); camilla.torreggiani@izsler.it (C.T.); benedetta.cordioli@izsler.it (B.C.); alice.prosperi@izsler.it (A.P.); andrea.luppi@izsler.it (A.L.); chiara.chiapponi@izsler.it (C.C.); 2 Biochemistry and Molecular Biology Unit, Department of Life Sciences, University of Parma, 43124 Parma, Italy

**Keywords:** swine influenza viruses, H3N2 viruses, reassortant strains, surveillance

## Abstract

In recent years, the four main swine influenza A virus (IAV-S) subtypes circulating in swine in the EU have been H1avN1, H1huN2, H1N1pdm09, and H3N2. The latter emerged in 1984 from a reassortment event between a human seasonal H3N2 and H1avN1, and is currently detected at low prevalence in swine in Italy. Here, we describe nine H3N2 IAV-S isolates belonging to three novel genotypes, first detected in Italy in 2021, likely resulting from reassortment events between swine and human IAVs. The first genotype was characterized by a hemagglutinin (H3 HA) of human seasonal origin, a neuraminidase (N2 NA) derived from H1huN2 strains circulating in Italian swine, and an avian-like internal gene cassette (IGC). The second genotype differed in its IGC constellation: PB2, PB1, PA and NP segments were of pandemic origin (pdm09), while NS and M segments derived from the Eurasian avian-like lineage. The third genotype combined a human-derived H3, a Gent/84-derived N2, and a pdm09-origin IGC, except for an avian-like NS. This study aimed to characterize the genetic features of these novel H3huN2 and assess their epidemiological relevance, with implications for surveillance and control, improving preparedness and mitigating the risks posed by zoonotic influenza viruses.

## 1. Introduction

Swine production has grown enormously worldwide over the past two decades, leading to structural changes in pig farms. Meanwhile, the dynamics of IAV-S infections have shifted from acute respiratory epizootic outbreaks to enzootic forms with recurrent circulation in many farms. Globally, the endemic subtypes of IAV-S circulating among pigs include H1N1, H1N2, and H3N2. However, the genetic diversity between these subtypes is enormous. In European pigs, the genetic and antigenic variability of IAV-S (H1N1, H1N2, H3N2, and H1N1pdm09) has significantly increased due to reassortment events and the emergence of antigenic variants [1,2].

Currently, influenza subtypes circulating among pigs in Europe and in Italy belong to distinct genetic lineages, dynamically evolving over time [3,4,5]. IAV-S subtypes are classified based on the combination of hemagglutinin (HA) and neuraminidase (NA) genes. Recently, porcine H1 HA sequences have been globally categorized into distinct genetic lineages [6]. Genetic variability is driven by bidirectional transmission events between humans and pigs, promoting host-specific viral adaptation and evolution [7,8].

The emergence of Eurasian avian-like (EA) H1avN1 viruses in European swine dates back to 1979 in Belgium and Germany [9]. These viruses, genetically distinct from the classical swine H1N1 lineage derived from the 1918 pandemic, arose from a triple reassortment event involving avian influenza viruses, with IAVs from ducks as ancestor strains [9,10]. The H1N1pdm09 virus, responsible for the 2009 global influenza pandemic, significantly impacted the European swine population, quickly spreading in pigs after human-to-swine transmission. This virus originated from a reassortment event between a North American triple reassortant virus (comprising genes derived from avian, human H3N2 and classical swine lineages), and the Eurasian avian-like H1N1 lineage, from which it acquired its NA and M gene segments [11]. The first reported outbreaks of H1N1pdm09 in European pigs occurred in 2009 in the United Kingdom and Ireland [12]. These initial incursions were attributed to multiple independent human-to-swine transmission episodes. Genotypic analyses confirmed the human origin of these swine isolates, which became established in European swine populations, reassorting with endemic IAV-S strains, leading to novel viral genotypes and further contributing to the genetic diversification of IAV-S [13].

The human-like H1N2 subtype (H1huN2) has become increasingly common in European pig populations, particularly in countries where H3N2 prevalence has decreased, underscoring the dynamic nature of influenza virus circulation in swine [7,14,15,16].

H3N2 influenza viruses have historically played a significant role in the epidemiology of IAV-S in Europe. While once widespread, their prevalence has decreased in several countries, including the United Kingdom, France, and Denmark, from the late 1980s to early 1990s, possibly due to competitive pressure from other subtypes, like H1huN2 and H1avN2 reassortants [16]. Nevertheless, H3N2 continues to circulate in Germany, Italy, Spain, and the Netherlands, suggesting regional variations in its circulation and the role of herd-specific factors [3,4,17]. While Italian H3N2 viruses may exhibit unique features, they are often genetically related to strains circulating in other European countries [3].

A significant portion of the observed IAV-S diversity results from reciprocal transmission between humans and swine, followed by viral changes caused by antigenic drift, and spread via the transport and trade of live animals [2,8,18]. The repercussions of these events were highlighted by the 2009 pandemic [19] and by ongoing introductions of the H1N1pdm09 virus into swine populations [19]. Additionally, human seasonal H3N2 spillovers into swine continue to be reported in recent years [7,19,20,21,22,23,24,25], with detection of swine H3 HA primarily paired with N2 NA lineages from the same initial human-to-swine spillover event [26]. The predominant contemporary H3 lineages found in pigs reflect the complex relationships of IAV among swine and humans. Swine lineages, from each decade starting from the 1970s, have been consistently circulating in global pig populations. Unlike the H1-1A lineage, most of these H3 lineages are limited in their geographic distribution, with the 1970.1 and 2000.3 clades being exclusively identified in Europe [7]. In 1984, reassortment between a human seasonal H3N2 virus with the H1avN1av subtype, with the former acquiring the IGC of the latter, led to the A/swine/Gent/1/1984-derived H3N2 lineage (H3-1970.1, N2g), which subsequently spread within the European and Italian swine population [4]. In Italy, the prevalence of this H3N2 lineage progressively declined in comparison to H1 subtypes, falling below 10% during 2020–2022. All the swine H3N2 strains sequenced in 2017–2020 in our laboratory fell within this genotype without evidence of reassortment events [3].

In Italy, we have described the variability of circulating IAV-S strains, which include H1 HAs of the H1-1A (pdm09-like), H1-1B (human-like), and H1-1C (avian-like) subtypes and related sub-clusters in combination with N1 (avian-like or pdm09) and N2 neuraminidase genes [3].

Here, we describe a newly identified H3N2 reassortant lineage circulating in Italian swine farms. Emerging after 2020, this lineage, characterized by a human-derived H3 HA segment, has shown active circulation and evidence of reassortment [3,27].

## 2. Materials and Methods

### 2.1. Sample Processing Workflow

Clinical samples—including oral fluids, nasal swabs, and lung tissues from necropsies—were obtained from respiratory outbreaks in pig farms in Northern Italy between January 2021 and March 2025. Samples that tested positive for IAV-S via real-time RT-PCR targeting of the M gene [28] were subtyped with nested RT-PCR [3].

Virus isolation was attempted from positive samples by inoculating susceptible cell cultures (MDCK and CACO-2) and 11-day-old SPF (specific-pathogen-free) embryonated chicken eggs [29]. Whole-genome sequencing (WGS) from clinical samples or available isolates was performed according to Lycett et al. [30]. DNA libraries were prepared with Nextera-XT DNA Library Preparation Kit (Illumina, Inc., San Diego, CA, USA), according to the manufacturer’s instructions. Libraries were quantified on a Quantus^TM^ fluorometer, diluted, pooled, and sequenced on a MiSeq^TM^ instrument (Illumina) using either the MiSeq Reagent Nano Kit v2 or the MiSeq Reagent Kit v2, in 2 × 150 bp paired-end runs. Raw sequencing reads were quality-filtered and de novo assembled using CLC Genomic Workbench v.11 (Qiagen, Milan, Italy).

### 2.2. Sequence Alignments and Phylogenetic Analyses

Phylogenetic analyses were performed on a dataset comprising European swine and human H3 HA and N2 NA sequences retrieved from NCBI’s GenBank. H3N2 and H1N2 European sequences of both swine and human origin were selected for analysis. All sequences used for phylogenetic analyses are listed in Supplementary File S2. Sequence alignments were performed using MAFFT [31], and manually refined with BioEdit software v7.2.5 [32] and AliView v1.28 [33]. Maximum Likelihood (ML) phylogenetic trees were inferred with Iqtree2 [34,35], and visualized with iTOL v7.2 [36]. Clade classification of H3 HA sequences was performed on the BV-BRC website using the Subspecies Classification section [37].

### 2.3. Map Representing the Swine Population in Italian Provinces

The map was created with RStudio (version 2023.12.0) with data updated as of 30 June 2024, obtained from the Italian Bank of Veterinary Data [38]. The following packages were used to create the map: rnaturalearth, rnaturalearthdata, sf, ggplot2, dplyr, viridis, readxl, and scales.

### 2.4. Hemagglutination Inhibition (HI) Tests

Hemagglutination inhibition (HI) assay was performed using turkey red blood cells, following the protocol outlined in the WOAH Manual of Diagnostic Tests and Vaccines for Terrestrial Animals, with the control virus A/swine/Italy/311349/2013 (H3 1970.1-N2g) and its homologous hyperimmune pig serum [39].

A total of 88 pig sera, previously collected for diagnostic purposes from influenza-vaccinated animals (CEVA Respiporc Flu3 and Respiporc Flu-Pan) on an Italian swine farm, were tested against A/swine/Italy/27326-07/2023/H3N2 (Other-Human-2010) and the reference Italian strain A/swine/Italy/311349/2013/H3N2, which was chosen as representative of the 1970.1 lineage in Italy, that had a hyperimmune serum previously produced in swine and is currently used in HI diagnostic tests in Italy.

## 3. Results

### 3.1. Virological Analysis

A total of 784 diagnostic swine samples, collected between January 2021 and March 2025 during 652 respiratory outbreaks on 359 pig farms in Northern Italy, tested positive for IAV by real-time RT-PCR and were subsequently characterized for IAV-S (Table 1). H3 HA-positive strains were detected at lower percentages each year compared with the other H1 HA subtypes. Interestingly, in 2025 we observed an increase in prevalence, reaching 13% in March, compared with the lower H3 prevalence recorded in previous years.

**Figure 1:**
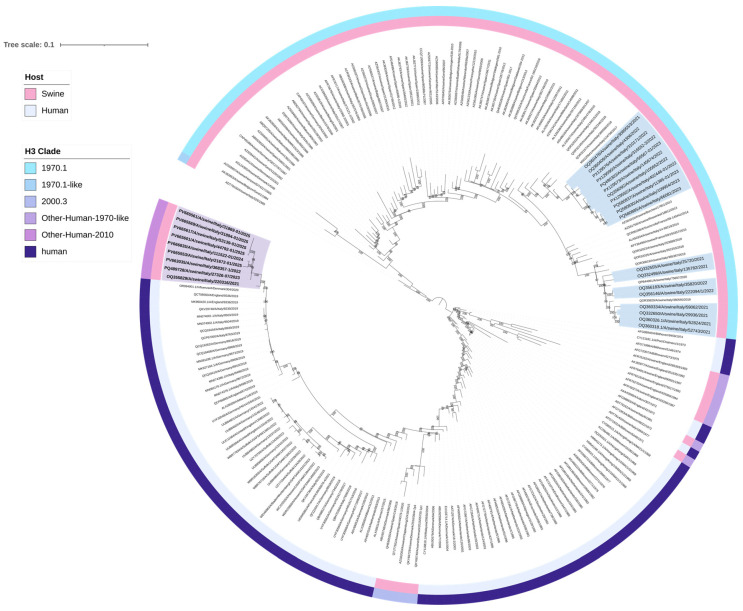
Maximum likelihood (ML) phylogenetic tree of hemagglutinin (H3 HA) genes from 201 swine and human H3N2 influenza viruses. The tree was visualized in iTOL version 7.2 and includes two annotation layers: the inner circle (closer to the tree tips) indicates host origin, distinguishing between swine and human, while the outer circle corresponds to clade classification. The nine human-like swine H3N2 belonging to the newly identified genotypes are in bold and highlighted in purple, clustering with human H3 HA sequences. The 19 H3 HA sequences belonging to clade 1970.1 are highlighted in light blue. Sequences are identified by their GenBank accession numbers. Alignment length: 1695 nucleotides; 1000 bootstrap replicates were performed to assess the robustness of the tree topology; bootstrap values below 75% are not shown. The scale bar represents the number of nucleotide substitutions per site.

**Figure 2:**
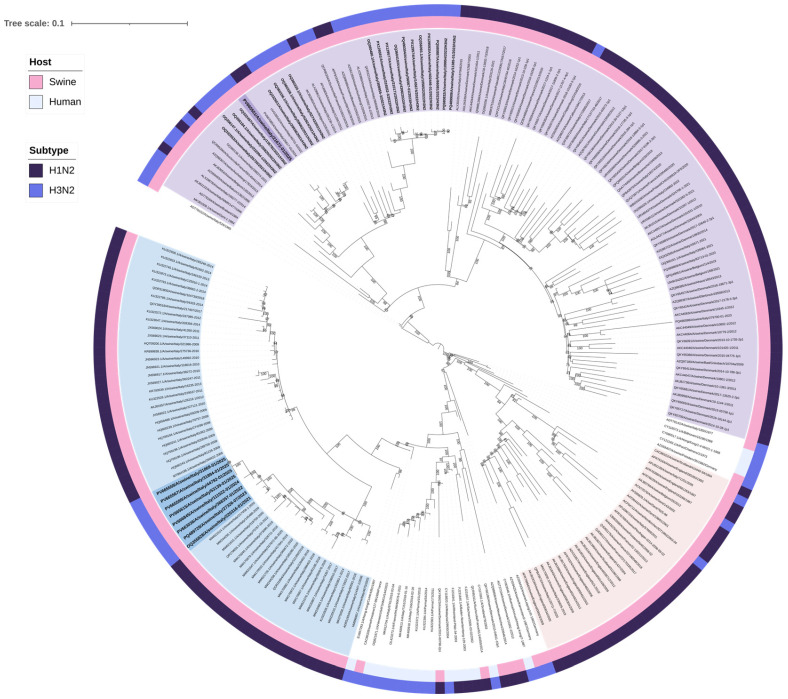
Maximum likelihood (ML) phylogenetic tree of neuraminidase (NA N2) genes from 223 swine and human H3N2 and H1N2 influenza viruses. The tree was visualized in iTOL version 7.2 and includes two annotation layers: the inner circle (closer to the tree tips) indicates host origin, distinguishing between swine and human, while the outer circle corresponds to the subtype. The nine H3N2 isolates belonging to the newly identified genotypes are shown in bold and are highlighted in blue (It-N2 lineage) and violet (N2g lineage). The N2 NAs of the 19 H3N2 belonging to the Gent/84-derived lineage are highlighted in bold. Sequences are identified by their GenBank accession number. Alignment length: 1389 nucleotides; 1000 bootstrap replicates were performed to assess the robustness of the tree topology; bootstrap values below 75% are not shown. Sequences color coding: violet-colored sequences represent the A/swine/Gent/1/1984-like derived NA lineage (N2g); light pink sequences represent A/swine/Scotland/410440/1994-like derived NA lineage (N2s); and blue sequences represent A/swine/Italy/4675/2003-like derived NA lineage (It-N2). The scale bar represents the number of nucleotide substitutions per nucleotide site.

A total of 28 H3N2 positive samples were sequenced (Table 2). BLAST (2.16.0) results confirmed the origin of internal gene segments from established swine lineages and/or pdm09-origin (Supplementary Tables S1–S9). Phylogenetic analysis of the H3 HA showed that nine swine H3N2 viruses, with H3 HA classified as clade “Other-Human-2010”, clustered closely with human H3 HA strains from Europe belonging to the 2019–2020 influenza season (Table 3, Figure 1). Eight of these nine “Other-Human-2010” H3N2 strains had an N2 gene clustering with NA sequences from an H1huN2 IAV-S genotype currently circulating in Italian swine since 2000 (It-N2 lineage) [3,5]. The strains detected up to 2023 had an avian-like IGC, while in 2024 and 2025, we detected two reassortant strains with different IGC combinations (avian-like and pdm09) (Table 3). One strain of this “Other-Human-2010” group carried a Gent/84-derived N2 NA (Table 3, Figure 2).

The other 19 H3N2 strains had H3 HA sequences belonging to clade 1970.1, clustering with swine sequences, N2 NA segments all belonging to the Gent/84-derived lineage (Figure 1 and Figure 2), and an avian-like internal gene cassette (IGC).

Interestingly, all the sequenced H3N2 strains of 2025 had the newly introduced “Other-Human-2010” H3 HA gene in combination with avian-like IGC and It-N2 neuraminidase.

**Figure 3:**
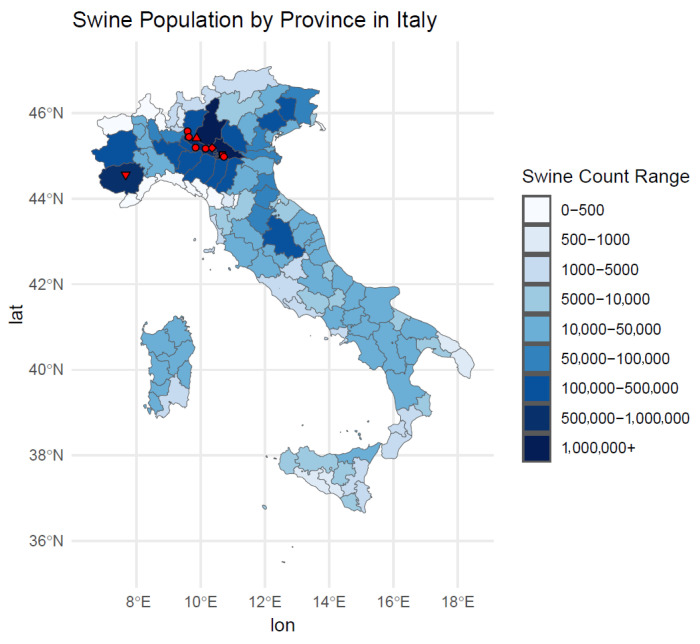
Map representing the swine population by province in Italy. Legend: the red triangle facing down corresponds to the 2021 isolate; the red diamond corresponds to the 2022 isolate; the red square corresponds to the 2023 isolate; the red triangle facing up corresponds to the 2024 isolate; red circles correspond to 2025 isolates.

Almost all the new lineage strains were detected in Northern Italy regions in areas with the highest pig density (Figure 3).

### 3.2. Serological Testing

To assess the antigenic reactivity of this new lineage, one of the H3N2 isolates, A/swine/Italy/27326-07/2023, was tested by HI using the reference hyperimmune serum produced in swine against the Italian isolate H3N2 A/swine/Italy/311349/2013. The antigen A/swine/Italy/27326-07/2023 was selected as it showed high amino acid similarity to the new H3N2 strains from 2024 and 2025 (>98%). Percentages of nucleotide and amino acid identity between this strain and the other eight H3N2 belonging to the new lineage are listed in Supplementary Table S11. The test revealed a low titer of 1:20 against the new strain, compared to a higher homologous titer of 1:640 observed for the reference serum-virus pair. Moreover, a group of 88 field sera, collected in 2023 for diagnostic purposes from an Italian pig farm, from animals vaccinated with both Respiporc Flu3 and Respiporc Flu-Pan influenza vaccine, were HI-tested against the two viruses. Eighty-six percent of the serum samples were positive (HI titer ≥20) against the swine-derived strain and negative against the newly detected human-derived H3N2 strain (Supplementary Table S10). Only six animals over 88 (6.8%) showed low reactivity titers to the new H3N2 lineage, in addition to positivity to the H3N2 1970.1 swine strain. These data could be explained by exposure of some of the animals to the new lineage.

## 4. Discussion

Here, we report the detection of nine reassortant swine H3N2 viruses characterized by an H3 HA gene segment derived from a human seasonal influenza virus (clade “Other-Human-2010”), combined with an N2 NA segment currently circulating among Italian swine (A/swine/Italy/4675/2003-like) and, in one instance, a Gent/84-derived N2 segment. These viruses also carry an IGC of EA and/or pdm09 origin. To our knowledge, these specific genetic constellations have not been previously reported in Italian swine IAV-S. Phylogenetic analysis showed that the H3 HA segments of these viruses cluster closely with European human H3N2 strains from 2019 to 2020, including Danish, Italian, German, and English isolates. It remains unclear whether these nine viruses originated from multiple independent events leading to the same genotypes, or from a single reassortment event followed by sustained viral circulation and detection between 2022 and 2025.

Since 2021, these reassortant H3N2 swine viruses have been sporadically detected in Northern Italy. Notably, the classical Gent/84-derived H3N2 genotype has been circulating at low prevalence in Italy over the past five years. The acquisition of a human-derived H3 HA gene may have provided an evolutionary advantage, enabling these reassortants to evade existing swine immunity, facilitating their spread among immunologically naïve pig populations [18,40]. To assess the antigenic relationship between these newly detected human-like H3N2 viruses and currently circulating swine strains, HI tests were performed. The test, performed using a reference H3 1970.1 hyperimmune antiserum against the A/swine/Italy/27326-07/2023 antigen, showed a very low HI titer (1:20), possibly suggesting no serological cross-reactivity.

Furthermore, the human-like H3N2 swine virus (A/swine/Italy/27326-07/2023) was tested against 88 recent sera, collected in 2023 from pigs vaccinated with commercial vaccines, and compared to a representative swine H3N2 strain (H3 1970.1-N2g). Most of the tested sera (93%) reacted with the H3 1970.1 reference antigen, while only 7% reacted with the new antigen A/swine/Italy/27326/7/2023.

Results showed no cross-reactivity between the two antigens, suggesting that antibodies induced by the vaccine, or by a previous exposure to a swine lineage H3N2 strain, failed to recognize the human-derived H3 HA. This highlights significant antigenic divergence between the human-like HA and the swine vaccine strain (A/swine/Germany/Bakum/IDT1769/2003 1970.1), raising concerns about the efficacy of existing vaccines in protecting pigs against such reassortant viruses, which may contribute to their ongoing circulation. The lack of herd immunity may also facilitate the circulation of the described lineage [24].

These viruses were detected primarily in areas with high swine population density in Italy, underscoring the link between intensive pig farming and heightened risk of viral reassortment.

The acquisition of a human-derived HA by a swine influenza virus suggests a potentially significant shift in viral infectivity and transmissibility among pigs. Notably, most of the detected reassortants also carry a human-derived N2 segment (It-N2), which was originally introduced into the swine population in the late 1990s. This generated a novel genetic makeup, combining a recent human-derived H3 HA with an older, established human-derived N2. Continuous surveillance is crucial to determine whether the prevalence of these emerging genotypes will increase or if those strains will remain sporadic. By acquiring the human H3, these viruses’ spread could be driven by selective advantages such as host immune escape, increased transmissibility, or overall better fitness [41]. Given the constantly evolving nature of IAVs, viral monitoring should be integrated into active surveillance plans, both at the farm level and also among swine industry personnel, as mixed infections could easily go undetected. Genomic analyses highlighted reassortment events between circulating H1N2 swine viruses and human H3 strains, underscoring the potential for genetic exchange between human and swine influenza IAVs. These reassortment events may contribute to the ongoing evolution and potential adaptation of swine influenza viruses, highlighting the importance of surveillance, particularly in areas with low but persistent circulation of specific subtypes [7].

## 5. Conclusions

We identified nine H3N2 IAV-S collected in Italy between 2021 and 2025, all carrying a human-derived H3 HA segment. These viruses belong to three distinct genotypes, each characterized by varying combinations of NA and internal gene segments of swine and pdm09 origin. Phylogenetic analysis suggests that the human H3 HA segment was introduced into the swine population via reassortment, potentially involving an endemic H1huN2 swine strain as the donor of the remaining gene segments. These findings highlight the ongoing risk for human-to-swine transmission and subsequent reassortment events, underscoring the need for sustained molecular surveillance at the human–animal interface to monitor and detect the emergence of novel genotypes with potential zoonotic implications.

## Figures and Tables

**Table 1:** Summary of IAV-S subtypes detected from January 2021 to March 2025 in IAV-positive samples. NT indicates non-typeable; x indicates a non-typeable segment (Hx or Nx).

Sampling Year	NT	H1Nx	H1N1	H1N2	H3Nx	H3N2	HxN1	HxN2	Outbreaks Analyzed n.
2021	15%	12.4%	31.1%	31.1%	1.6%	3.1%	2.1%	4.1%	193
2022	15%	11.4%	34.3%	25.9%	1.2%	6%	3%	3%	166
2023	8%	6.3%	25%	50%	0%	7.1%	0.9%	2.7%	112
2024	11%	10.6%	31.7%	42.3%	0.7%	1.4%	0.7%	1.4%	142
2025 (March)	5.1%	5.1%	38.5%	28.2%	0%	12.8%	2.6%	2.6%	39

**Table 2:** H3 HA lineages of the 28 sequenced H3N2 strains characterized from 2021 to 2025. The number of sequences and their relative proportion are shown for each year.

Year	H3 (1970.1)		Other-Human-2010		Total Analyzed
2021	87.5%	7	12.5%	1	8
2022	89%	8	11%	1	9
2023	50%	1	50%	1	2
2024	75%	3	25%	1	4
2025	0%	0	100%	5	5

**Table 3:** Overview of the nine H3N2 swine strains belonging to the new genotypes. H3 HA clades were classified according to https://www.bv-brc.org/app/SubspeciesClassification [37]. NA lineages were assigned as previously described: N2g (A/swine/Gent/1/1984-like), It-N2 (A/swine/Italy/4675/2003-like), EA (Eurasian avian-like) [3].

ID	Sampling Date	Italian Region	H3 HA	NA	PB2	PB1	PA	NP	M	NS	Sampling Year
A/swine/Italy/220316/2021	25 June 2021	Piemonte	Other-Human-2010	It-N2	EA	EA	EA	EA	EA	EA	2021
A/swine/Italy/368357-01/2022	9 November 2022	Lombardia	Other-Human-2010	It-N2	EA	EA	EA	EA	EA	EA	2022
A/swine/Italy/27326-07/2023	25 January 2023	Lombardia	Other-Human-2010	It-N2	EA	EA	EA	EA	EA	EA	2023
A/swine/Italy/111522-01/2024	9 April 2024	Lombardia	Other-Human-2010	It-N2	pdm	pdm	pdm	pdm	EA	EA	2024
A/swine/Italy/21672-01/2025	23 January 2025	Emilia-Romagna	Other-Human-2010	N2g	pdm	pdm	pdm	pdm	pdm	EA	2025
A/swine/Italy/44792-01/2025	14 February 2025	Lombardia	Other-Human-2010	It-N2	EA	EA	EA	EA	EA	EA	2025
A/swine/Italy/31869-01/2025	3 February 2025	Lombardia	Other-Human-2010	It-N2	EA	EA	EA	EA	EA	EA	2025
A/swine/Italy/31894-01/2025	4 February 2025	Lombardia	Other-Human-2010	It-N2	EA	EA	EA	EA	EA	EA	2025
A/swine/Italy/53139-01/2025	24 February 2025	Lombardia	Other-Human-2010	It-N2	EA	EA	EA	EA	EA	EA	2025

## Data Availability

The sequences of the strains object of this study are available in GenBank, accession numbers: OQ355822-OQ355829; PV663029-PV663036; PQ489728-PQ489732, PQ489922, PQ489976, PQ490019; PV666835-PV666842; PV665546-PV665553; PV665594-PV665601; PV665554-PV665561; PV665562-PV665569; PV665610-PV665617; OQ332548-OQ332555; OQ332644-OQ332651; OQ332493-OQ332500; PX129568-PX129575; PX129584-PX129591; PX129576-PX129583; PX129592-PX129599; PQ490303-PQ490310; PQ580837-PQ580844; PQ580885-PQ580892; PQ580830-PQ580836.

## Supplementary Materials

The following supporting information can be downloaded at: https://www.mdpi.com/article/10.3390/v17091171/s1, Supplementary File S1: Supplementary Tables S1–S11. Supplementary File S2: datasets of H3 HA and N2 NA sequences used in this study.
