# Supplementary material for: Human-Derived H3N2 Influenza A Viruses Detected in Pigs in Northern Italy
Source: Viruses. 2025 Aug 27;17(9):1171. doi: 10.3390/v17091171 (PMC12474197; doi:10.3390/v17091171)
Supplement: Supplementary file 1 [file viruses-17-01171-s001.zip › Supplementary File S1_.pdf]

Supplementary Tables S1-S9. Closest sequences detected with BLASTn for the internal gene cassette (IGC) segments of the 9 H3N2 viruses object of analysis.

Supplementary Table S1. Highest scoring matches in BLASTn for sample A/swine/Italy/220316/2021. Blast search performed on 2025-05-21.

	**A/swine/Italy/220316/2021**		
**Segment**	Strain name	Percent Identity	Accession number
**PB2**	A/swine/Italy/217458-1/2020(H1N2)	99.43%	MW621519.1
**PB1**	A/swine/Italy/217458-1/2020(H1N2)	99.34%	MW621518.1
**PA**	A/swine/Italy/217458-1/2020(H1N2)	99.30%	MW621517.1
**NP**	A/swine/Italy/57984/2019(H1N2)	99.47%	MW169976.1
**M**	A/swine/Italy/236197/2018(H1N1)	99.59%	MW171086.1
**NS**	A/swine/Italy/57984/2019(H1N2)	99.52%	MW169532.1

Supplementary Table S2. Highest scoring matches in blast for sample A/swine/Italy/368357-01/2022. Blast search performed on 2025-05-21.

	**A/swine/Italy/368357-01/2022**		
**Segment**	Strain name	Percent Identity	Accession number
**PB2**	A/swine/Italy/IZSLER-6097-20/2024(H1N2)	99.61%	PP864068.1
**PB1**	A/swine/Italy/IZSLER-6097-20/2024(H1N2)	99.69%	PP864069.1
**PA**	A/swine/Italy/IZSLER-6097-20/2024(H1N2)	99.44%	PP864070.1
**NP**	A/swine/Italy/IZSLER-6097-20/2024(H1N2)	99.67%	PP864072.1
**M**	A/swine/Italy/IZSLER-6097-20/2024(H1N2)	99.69%	PP864073.1
**NS**	A/swine/Italy/57984/2019(H1N2)	99.05%	MW169532.1

Supplementary Table S3. Highest scoring matches in blast for sample A/swine/Italy/27326-07/2023. Blast search performed on 2025-05-21.

	**A/swine/Italy/27326-07/2023**		
**Segment**	Strain name	Percent Identity	Accession number
**PB2**	A/swine/Italy/IZSLER-6097-20/2024(H1N2)	99.21%	PP864068.1
**PB1**	A/swine/Italy/IZSLER-6097-20/2024(H1N2)	99.47%	PP864069.1
**PA**	A/swine/Italy/IZSLER-6097-20/2024(H1N2)	99.02%	PP864070.1
**NP**	A/swine/Italy/IZSLER-6097-20/2024(H1N2)	99.53%	PP864072.1
**M**	A/swine/Italy/248971/2019(H1N2)	99.49%	MW170224.1
**NS**	A/swine/Italy/57984/2019(H1N2)	99.05%	MW169532.1

Supplementary Table S4. Highest scoring matches in blast for sample A/swine/Italy/111522-01/2024. Blast search performed on date 2025-05-21.

	**A/swine/Italy/111522-01/2024**		
**Segment**	Strain name	Percent Identity	Accession number
**PB2**	A/swine/Denmark/20-3797-4/2020(H1N1)	98.11%	PQ193743.1
**PB1**	A/swine/Denmark/S19922-5/2021(H1N1)	98.68%	ON716252.1
**PA**	A/swine/Denmark/S19922-5/2021(H1N1)	98.65%	ON716253.1
**NP**	A/swine/Denmark/S19922-5/2021(H1N1)	99.06%	ON716255.1
**M**	A/swine/Italy/IZSLER-6097-20/2024(H1N2)	99.59%	PP864073.1
**NS**	A/swine/Italy/426696/2022(H1N2)	98.57%	PQ535235.1

Supplementary Table S5. Highest scoring matches in blast for sample A/swine/Italy/21672-01/2025. Blast search performed on date 2025-05-21.

	**A/swine/Italy/21672-01/2025**		
**Segment**	Strain name	Percent Identity	Accession number
**PB2**	A/swine/Denmark/20-3797-4/2020(H1N1)	98.07%	PQ193743.1
**PB1**	A/swine/Denmark/S19922-5/2021(H1N1)	98.42%	ON716252.1
**PA**	A/swine/Denmark/S19922-5/2021(H1N1)	98.09%	ON716253.1
**NP**	A/swine/Denmark/S19922-5/2021(H1N1)	99.00%	ON716255.1
**M**	A/swine/Italy/391086/2020(H1N1)	99.08%	MZ404277.1
**NS**	A/swine/Italy/426696/2022(H1N2)	98.33%	PQ535235.1

Supplementary Table S6. Highest scoring matches in blast for sample A/swine/Italy/31869-01/2025. Blast search performed on date 2025-05-21.

	**A/swine/Italy/31869-01/2025**		
**Segment**	Strain name	Percent Identity	Accession number
**PB2**	A/swine/Italy/IZSLER-6097-20/2024(H1N2)	98.68%	PP864068.1
**PB1**	A/swine/Italy/IZSLER-6097-20/2024(H1N2)	99.12%	PP864069.1
**PA**	A/swine/Italy/IZSLER-6097-20/2024(H1N2)	98.51%	PP864070.1
**NP**	A/swine/Italy/IZSLER-6097-20/2024(H1N2)	99.20%	PP864072.1
**M**	A/swine/Italy/IZSLER-6097-20/2024(H1N2)	99.59%	PP864073.1
**NS**	A/swine/Italy/57984/2019(H1N2)	98.33%	MW169532.1

Supplementary Table S7. Highest scoring matches in blast for sample A/swine/Italy/31894-01/2025. Blast search performed on date 2025-05-21.

	**A/swine/Italy/31894-01/2025**		
**Segment**	Strain name	Percent Identity	Accession number
**PB2**	A/swine/Italy/IZSLER-6097-20/2024(H1N2)	98.68%	PP864068.1
**PB1**	A/swine/Italy/IZSLER-6097-20/2024(H1N2)	99.12%	PP864069.1
**PA**	A/swine/Italy/IZSLER-6097-20/2024(H1N2)	98.42%	PP864070.1
**NP**	A/swine/Italy/IZSLER-6097-20/2024(H1N2)	99.20%	PP864072.1
**M**	A/swine/Italy/IZSLER-6097-20/2024(H1N2)	99.59%	PP864073.1
**NS**	A/swine/Italy/57984/2019(H1N2)	98.33%	MW169532.1

Supplementary Table S8. Highest scoring matches in blast for sample A/swine/Italy/44792-01/2025. Blast search performed on date 2025-05-21.

	**A/swine/Italy/44792-01/2025**		
**Segment**	Strain name	Percent Identity	Accession number
**PB2**	A/swine/Italy/IZSLER-6097-20/2024(H1N2)	98.68%	PP864068.1
**PB1**	A/swine/Italy/IZSLER-6097-20/2024(H1N2)	99.16%	PP864069.1
**PA**	A/swine/Italy/IZSLER-6097-20/2024(H1N2)	98.61%	PP864070.1
**NP**	A/swine/Italy/IZSLER-6097-20/2024(H1N2)	99.20%	PP864072.1
**M**	A/swine/Italy/IZSLER-6097-20/2024(H1N2)	99.49%	PP864073.1
**NS**	A/swine/Italy/57984/2019(H1N2)	98.33%	MW169532.1

Supplementary Table S9. Highest scoring matches in blast for sample A/swine/Italy/53139-01/2025. Blast search performed on date 2025-05-21.

	**A/swine/Italy/53139-01/2025**		
**Segment**	Strain name	Percent Identity	Accession number
**PB2**	A/swine/Italy/IZSLER-6097-20/2024(H1N2)	98.68%	PP864068.1
**PB1**	A/swine/Italy/IZSLER-6097-20/2024(H1N2)	99.03%	PP864069.1
**PA**	A/swine/Italy/IZSLER-6097-20/2024(H1N2)	98.47%	PP864070.1
**NP**	A/swine/Italy/IZSLER-6097-20/2024(H1N2)	99.20%	PP864072.1
**M**	A/swine/Italy/IZSLER-6097-20/2024(H1N2)	99.59%	PP864073.1
**NS**	A/swine/Italy/57984/2019(H1N2)	98.33%	MW169532.1

Supplementary Table S10. HI titers of 88 field swine serum samples, collected in 2023, tested using the swine H3N2 lineage A/swine/Italy/311349/2013 and the human-derived clade A/swine/Italy/27326-07/2023. The reactivity of the hyperimmune serum A/swine/Italy/ 311349/2013 is shown in the last row.

	Virus	
Serum sample ID	A/swine/Italy/ 311349/2013-sw	A/swine/Italy/27326/7/2023-hu
1	20	NEG
2	20	NEG
3	20	NEG
4	40	NEG
5	20	NEG
6	40	NEG
7	80	NEG
8	80	NEG
9	80	NEG
10	20	NEG
11	NEG	NEG
12	20	NEG
13	NEG	NEG
14	NEG	NEG
15	20	NEG
16	40	NEG
17	40	NEG
18	80	NEG
19	40	NEG
20	320	NEG
21	320	NEG
22	80	NEG
23	40	NEG
24	80	NEG
25	160	NEG
26	160	NEG
27	80	NEG
28	40	NEG
29	40	NEG
30	40	NEG

31	40	NEG
32	40	NEG
33	80	NEG
34	80	NEG
35	160	NEG
36	160	NEG
37	160	NEG
38	80	NEG
39	160	NEG
40	80	NEG
41	80	NEG
42	640	NEG
43	640	NEG
44	320	NEG
45	320	NEG
46	NEG	NEG
47	NEG	NEG
48	80	80
49	160	NEG
50	320	NEG
51	NEG	NEG
52	640	20
53	160	NEG
54	160	NEG
55	160	NEG
56	160	NEG
57	80	NEG
58	160	NEG
59	160	20
60	160	NEG
61	160	NEG
62	40	40
63	160	NEG
64	320	NEG
65	160	NEG
66	40	NEG
67	40	NEG
68	80	NEG
69	80	NEG
70	80	NEG
71	80	NEG
72	320	NEG
73	320	NEG
74	320	NEG
75	80	NEG
76	160	NEG

77	160	NEG
78	40	NEG
79	160	80
80	640	NEG
81	320	NEG
82	160	80
83	160	NEG
84	320	NEG
85	80	NEG
86	80	NEG
87	320	NEG
88	160	NEG
A/swine/Italy/311349/2013-sw	640	20

Supplementary Table S11

Percentages of nucleotide and amino acid identities between strain A/swine/Italy/27326-07/2023 and the other eight H3N2 strains representing the new genotypes.

**Strain**	**Nucleotide identity (%) to A/swine/Italy/27326-07/2023**	**Amino acid identity (%) to A/swine/Italy/27326-07/2023**
A/swine/Italy/220316-01/2021	98.41%	97.70%
A/swine/Italy/368357-1/2022	99.88%	100.00%
A/swine/Italy/111522-01/2024	99.24%	99.29%
A/swine/Italy/21672-01/2025	98.82%	99.65%
A/swine/Italy/31869-01/2025	99.18%	99.12%
A/swine/Italy/31894-01/2025	99.18%	99.12%
A/swine/Italy/44792-01/2025	99.06%	98.76%
A/swine/Italy/53139-01/2025	99.12%	99.12%
